# Therapeutic Potential of 5′-Methylschweinfurthin G in Merkel Cell Polyomavirus-Positive Merkel Cell Carcinoma

**DOI:** 10.3390/v14091848

**Published:** 2022-08-23

**Authors:** Emily J. Koubek, Jillian S. Weissenrieder, Luz E. Ortiz, Nnenna Nwogu, Alexander M. Pham, J. Dylan Weissenkampen, Jessie L. Reed, Jeffrey D. Neighbors, Raymond J. Hohl, Hyun Jin Kwun

**Affiliations:** 1 Department of Medicine, Penn State College of Medicine, Hershey, PA 17033, USA; 2 Department of Pharmacology, Penn State College of Medicine, Hershey, PA 17033, USA; 3 Penn State Cancer Institute, Hershey, PA 17033, USA; 4 Department of Microbiology and Immunology, Penn State College of Medicine, Hershey, PA 17033, USA; 5 Department of Genetics, University of Pennsylvania, Philadelphia, PA 19104, USA

**Keywords:** schweinfurthin, Merkel cell carcinoma, Merkel cell polyomavirus, small T, PI3K/AKT, MAPK/ERK

## Abstract

Merkel cell carcinoma (MCC) is a rare but aggressive form of skin cancer predominantly caused by the human Merkel cell polyomavirus (MCPyV). Treatment for MCC includes excision and radiotherapy of local disease, and chemotherapy or immunotherapy for metastatic disease. The schweinfurthin family of natural compounds previously displayed potent and selective growth inhibitory activity against the NCI-60 panel of human-derived cancer cell lines. Here, we investigated the impact of schweinfurthin on human MCC cell lines. Treatment with the schweinfurthin analog, 5′-methylschweinfurth G (MeSG also known as TTI-3114), impaired metabolic activity through induction of an apoptotic pathway. MeSG also selectively inhibited PI3K/AKT and MAPK/ERK pathways in the MCPyV-positive MCC cell line, MS-1. Interestingly, expression of the MCPyV small T (sT) oncogene selectively sensitizes mouse embryonic fibroblasts to MeSG. These results suggest that the schweinfurthin family of compounds display promising potential as a novel therapeutic option for virus-induced MCCs.

## 1. Introduction

In recent years, there has been a surge in the development of a new class of anticancer drugs that lack the toxicity and chemoresistance of chemotherapeutic agents currently available [1]. Historically, natural products derived from plants and animals have been the source of all medicinal preparations. Recently, there has been an increased emphasis on developing natural products as anticancer drugs [1]. As of 2014, a total of 136 anticancer drugs were available worldwide, of which 83% were either natural products or synthesized to imitate natural products, and 17% were of synthetic origin [1]. The efficacy of developing natural products for chemotherapeutic purposes was famously observed in the antineoplastic drug, paclitaxel. Having as its primary objective the development of novel, potent and less toxic drugs, pharmaceutical research has focused on plant-based drug discovery. Approximately 1000 unique plant species have been reported to have anticancer potential [2]. 

Schweinfurthins are a small class of molecules isolated from an African plant of genus *Macaranga* [3]. This genus includes more than 300 species and has yielded many medicinal products, including but not limited to antibacterial prenylated flavonones, chromenoflavones and diterpenes. Members of this family were found to display potent anticancer activity against the NCI-60 cancer cell panel [3,4]. Since their initial identification as potential anticancer agents, a total of seventeen compounds schweinfurthin A-Q have been isolated from the *Macaranga* family, of which 12 show significant anticancer effects in cell culture [3,5]. Due to difficulties obtaining sufficient active materials from the plant, synthetic analogs of schweinfurthins were developed to model the same cytotoxicity and pattern of activity as the natural products [6,7,8]. Moreover, these synthetic analogs have increased compound stability [9]. 

The activity of Schweinfurthin analog, MeSG, has been studied in various human cancer cell lines. Two compounds, TTI-4242 and MeSG, have been shown to reduce cell proliferation in human melanoma cell lines, and tumor progression in a B16.F10 skin melanoma in vivo mouse model [10]. Additionally, in Swarm rat chondrosarcoma (SRC), a chemo resistant form of bone cancer, the efficacy of MeSG has been observed where treated mice displayed a significant decrease in tumor cell proliferation and increased cell death in comparison to untreated mice [11]. Therefore, it is essential to further investigate the feasibility of utilizing MeSG as a tumor suppressor agent in other aggressive chemo resistant cancers.

Merkel cell carcinoma (MCC) is a rare but highly aggressive form of skin cancer of neuroendocrine origin [12]. Although the incidence rate of MCC is significantly lower than melanoma [13], it is twice as deadly as malignant melanoma due to its high propensity to migrate and metastasize [14]. MCC has an estimated mortality rate of 33–46% and has since become the second leading cause of skin cancer-related deaths [15,16]. In most MCC cases (~90%), primary tumors are located around the head and neck regions as well as other sun-exposed skin areas, suggesting that UV exposure plays a critical role in MCC development [17,18]. Existing epidemiological data has defined risk factors for MCC, which include advanced age, a compromised immune system due to immunocompromising illnesses or organ transplant, and UV exposure [12]. 

The majority of MCC cases (~80%) have been linked to Merkel cell polyomavirus (MCPyV) [19], whereas the virus-negative cases contain single nucleotide polymorphisms consistent with UV radiation, which results in the observed high mutational burden and their tendency to appear in areas typically exposed to the sun [20,21,22]. MCPyV is a common skin commensal acquired as an asymptomatic childhood infection. Like other classic polyomaviruses, MCPyV is a small double-stranded DNA virus that expresses differentially spliced early mRNAs encoding small and large tumor (sT and LT) antigens that are essential for viral pathogenesis [23]. MCPyV sT, the main oncoprotein of MCPyV, is sufficient to induce loss of contact inhibition, anchorage independence, serum independent growth, and rodent fibroblast transformation as demonstrated by in vitro and in vivo studies [24,25,26,27]. MCPyV sT promotes stabilization of cellular oncogenes through its unique disordered LT stabilization domain (LSD) by impairing the activity of the E3 ubiquitin ligase Fbw7 [27,28], which potentially plays a vital role in regulating MCPyV sT-induced transformation [24,27], genomic instability [29], epithelial-to-mesenchymal transition (EMT) and cell migration [30], and NF-kB signaling to promote tumorigenesis [31]. Additionally, MCPyV sT promotes hyper-phosphorylation of the translational repressor 4E-BP1 and inhibits phosphatase activity of PP2A [26,32], which plays a critical role in activating mammalian target of rapamycin (mTOR) pathway for cell growth [26,33]. 

Currently, treatment of metastasized MCC involves broad-spectrum chemotherapy. However, response rates are relatively weak (52–61%), and significant treatment-related toxicity is seen, especially in older patients [34,35]. Studies have shown that 5′-methylschweinfurthin G (MeSG, TTI-3114) regulates the oncogenic PI3K/AKT/mTOR signaling pathway [36]. Activation of PI3K/AKT/mTOR pathway is frequently detected in MCC [37,38], which implicates TTI-3114 as a potential treatment option for advanced MCC. In this study, we examined the potential therapeutic efficacy of TTI-3114 in MCC.

## 2. Materials and Methods

### 2.1. Cell Culture and Lentiviral Transduction

MCC13 (RRID:CVCL_2583) and MKL-1 (RRID:CVCL_2600) cell lines were obtained from ATCC (Manassas, VA, USA). MKL-2 (RRID:CVCL_D027) and MS-1 (RRID:CVCL_E995) cells were generous gifts from Chang-Moore lab (University of Pittsburgh). MCC13, MS-1 and MKL-2 cells were cultured in RPMI-1640 supplemented with 15% FBS (Thermo Fisher Scientific, Waltham, MA, USA). MKL-1 cells were grown in RPMI-1640 supplemented with 10% FBS. All cell media was supplemented with penicillin, streptomycin, and Gibco Amphotericin B (Thermo Fisher Scientific). NIH3T3 cells stably expressing MCPyV sT antigens [28] were grown in DMEM with 5% bovine calf serum (Seradigm, Providence, UT, USA). Cells were maintained at 37 °C and 5% CO_2_ and used within 25 passages. MeSG (TTI-3114) was obtained from Terpenoid Therapeutics, Inc. (Coralville, IA, USA). For NIH3T3 transduction, lentiviral constructs were transfected into 293FT cells (Invitrogen, Carlsbad, CA, USA) with lentiviral constructs and packaging plasmids, psPAX2 and pMD2.G (Addgene, Watertown, MA, USA) using jetOPTIMUS (Polyplus, New York, NY, USA) according to the manufacturer’s instructions. NIH3T3 cells were transduced with lentivirus encoding pLVX empty vector, MCPyV sT.wt, MCPyV sT.LSDm [28] in the presence of 6 µg/mL polybrene (Sigma-Aldrich, St. Louis, MO, USA) and followed by selection with puromycin (3 μg/mL). 

### 2.2. MTT Assay

Cells were seeded at 15,000 cells/well (MKL-1, MKL-2, MS-1) or 5000 cells/well (MCC13) in 96-well plates. The next day, cells were incubated with indicated concentrations of MeSG at 37 °C and 5% CO_2_. After 44 h (h), 10 μL of 5 mg/mL MTT salt (Invitrogen, Thermo Fisher Scientific) was added to each well. Plates were returned to cell incubators for an additional four h. MTT salt reduction was terminated by addition of stop solution (80% isopropanol, 10% 1 N HCl, 10% Triton X-100), and plates were incubated at 37 °C overnight. The next day, the optical density of each well was measured at 540 nM and 650 nM (reference wavelength) using a SpectraMax i3X plate reader (Molecular Devices, San Jose, CA, USA). The absorbance is reported as the difference between the test (540 nM) and reference wavelength (650 nM) and is displayed as percent of control. 

### 2.3. Apoptosis Assay by Flow Cytometry Using Annexin V and 7-AAD Staining

Cells were incubated in the presence or absence of 100 nM MeSG. After 48 h, cells were collected and washed twice with cold PBS (Thermo Fisher Scientific, Waltham, MA USA). Cells were grown in suspension (MKL-1 and MS-1) and incubated with 1× accutase (Thermo Fisher Scientific) to break up aggregates and washed again with cold PBS. Cells were then re-suspended in 1× Annexin V Binding Buffer (BD Biosciences, San Jose, CA, USA) and incubated with PE Annexin V and 7-AAD (BD Biosciences) at room temperature for 15 min in the dark. Samples were then analyzed using a BD Biosciences FACS Fortessa 16 in the Penn State College of Medicine Flow Cytometry Core Facility, and data was analyzed using FlowJo software 10.7.1 (Tree Star, Ashland, OR, USA). 

### 2.4. Migration and Invasion Assays

Migration and invasion transwell assays were carried out using a CytoSelect™ cell migration and invasion assay kit (Cell Biolabs Inc., San Diego, CA, USA) according to the manufacturer’s instructions. Migration inserts contained a polycarbonate membrane with 8 μm pores, while invasion inserts contained a polycarbonate membrane coated with a dried basement membrane matrix solution. Cells in serum-free media were seeded at 1 × 10^6^ cells into inserts in a 24-well plate. MeSG was added to the cell suspension at a concentration of 0, 3, 10, or 30 nM. Media containing 10% (MKL-1) or 20% (MS-1, MKL-2, MCC13) FBS was added to the well outside the insert to act as a chemoattractant. Cells were incubated at 37 °C and allowed to migrate for 24 h or invade for 48 h. Following TTI-3114 incubation, cotton swabs were used to remove the non-migratory or non-invasive cells that remained inside the insert. The insert was stained for 10 min at room temperature with cell stain solution and then rinsed with water. Dried inserts were moved to an empty well, and the stain was eluted by gentle agitation for 10 min with an extraction solution. To quantify migration and invasion, eluted samples were moved to a 96-well plate and the optical density of each sample was measured at 560 nm using a SpectraMax i3X plate reader (Molecular Devices). Results are presented as percent of vehicle control values. 

### 2.5. Cell Viability Assay 

2 × 10^3^ cells/well (NIH3T3 cell line) were seeded in triplicate in a 96-well plate with complete growth medium. Cell proliferation/viability was measured using a CCK-8 assay kit (Sigma-Aldrich). Absorbance at 450 nm was measured using a Microtiter plate reader (Promega, Madison, WI, USA).

### 2.6. The 3D Tumor Spheroid Assay 

NIH3T3 cells expressing MCPyV sT were trypsinized and incubated with magnetic NanoShuttle-PL (Greiner Bio-One, Kremsmünster, Austria) for 24 h. NanoShuttle-PL magnetizes cells by electrostatically attaching to cell membranes during an overnight static incubation. Cells were counted (1 × 10^4^ cells/well) and seeded in triplicate in a 24-well cell-repellent surface plate. Magnetized cells were aggregated with magnetic tools to form spheroids for 24 h and subsequently cultured in DMEM medium containing 5% BCS for 10 days after MeSG treatments. Then images were taken using a REVOLVE4 microscope Echo Pro software (Echo Laboratories, San Diego, CA, USA) to measure the size of the spheroids.

### 2.7. RNA-Seq Analysis and Protein Detection

MS-1 vehicle and MeSG-treated samples (1 × 10^6^) were harvested at 48 h post TTI-3114 (100 nM) treatment. The experiments were done in duplicate. RNA sequencing and gene reads were aligned to the GRCh38.p13 human genome. Raw gene counts were uploaded to the integrated Differential Expression and Pathway (iDEP) website analysis and were transformed and normalized using the iDEP workflow [39] (Supplementary Materials). All gene counts below 0.5 counts per million (CPM) were excluded from the analysis. The 2000 most variable genes were subjected to k-means clustering (4 clusters) and pathway enrichment analysis using the gene ontology (GO) molecular function database. Differential expressed gene (DEG) analysis was conducted using DESeq2. The top upregulated or downregulated DEGs were identified using the following parameters: false-discovery rate (FDR) cutoff of 0.1 (FDR < 0.1) and a minimum fold change of 2. The top DEGs were run through KEGG and ShinyGO enriched pathway analyses using the default settings [40]. All figures were generated from iDEP or ShinyGo. Significantly differentially expressed genes were also compared to those identified by previously published work by Weissenrieder et al. [41] in sensitive SF-295 and resistant A549 cells treated with the synthetic schweinfurthin analogue, TTI-3066. Genes were filtered for significance and similar directionality, then correlations for log fold change vs. beta value were obtained with R (Supplementary Materials). MS-1 cells were treated with MeSG (1 μM) for 48 h to detect protein levels. Primary antibodies used in this study included 2B4 (Santa Cruz Biotechnology, sc-136172), 2T2 (MilliporeSigma, MABF2316), AKT (pan) (Cell Signaling, C67E7), Phospho-AKT (Ser473) (Cell Signaling, D9E), Phospho-S6 Ser235/236 (Cell Signaling, D57.2.2E), Phospho-p44/42 MAPK (Erk1/2) Thr202/Tyr204 (Cell Signaling, D13.14.4E), Phospho-p90RSK Ser380 (Cell Signaling, D3H11), and β-Actin (Cell Signaling, 13E5). All signals were detected with quantitative Infrared (IR) secondary antibodies (IRDye 800CW goat anti-mouse, 800 CW goat anti-rabbit, 680 LT goat anti-rabbit IgG, 680 LT goat anti- mouse IgG) (LI-COR) using a quantitative Infrared (IR) imaging system, Odyssey CLX (LI-COR Biosciences, Lincoln, NE, USA).

## 3. Results

### 3.1. MeSG Inhibits MCC Cell Metabolic Activity

**Figure 1:**
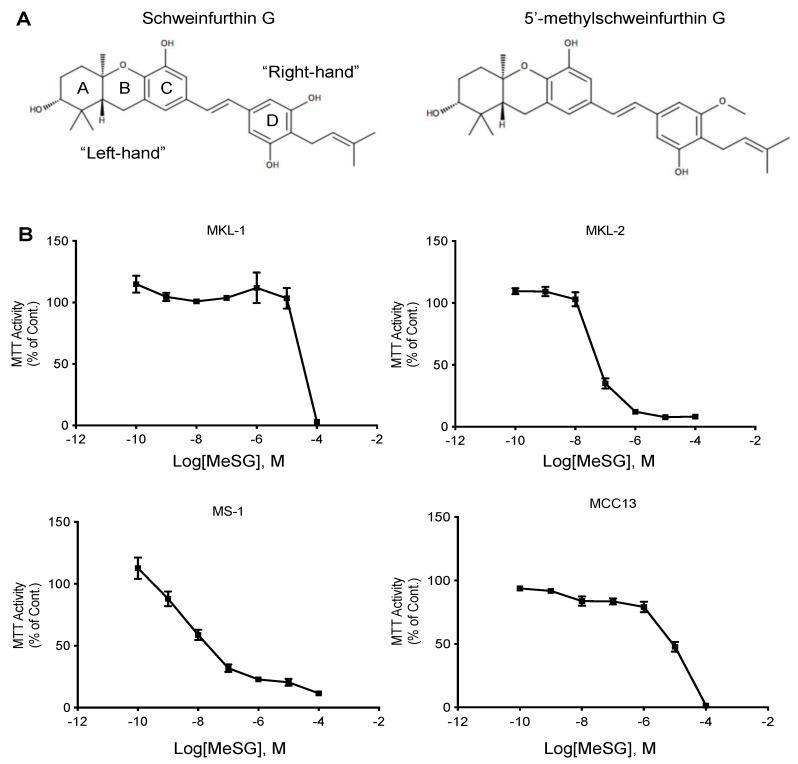
MeSG impairs MTT activity of human Merkel cell carcinoma cell lines. (**A**) Structures of the natural schweinfurthin G and the synthetic schweinfurthin analog MeSG, TTI-3114. (**B**) The human Merkel cell carcinoma cell lines MKL−1, MKL-2, MS-1, and MCC13 were incubated with increasing doses of MeSG (100 pM-100 μM) for 48 h. The impact of MeSG treatment on MTT activity was assessed by MTT assay. Data are displayed as a percentage of control and are representative of three independent experiments (*n* = 3, mean ± SEM).

To assess the cytotoxic effects of schweinfurthin in MCC cell lines, MTT assays were performed. Four human MCC cell lines were assessed: three MCPyV-positive (VP-MCC) cell lines (MKL-1, MKL-2, and MS-1) and one MCPyV-negative (VN-MCC) cell line (MCC13). These cell lines were not previously assessed in the preliminary NCI-60 cell panel screening and thus have been tested against a synthetic analog [42], TTI-3114. TTI-3114 includes a methylation on the 5′ phenol group of the D-ring (Figure 1A). MCC cells were incubated for 48 h with TTI-3114, and the cell metabolic activity was measured by MTT assay (Figure 1B). Results demonstrated that the MS-1 and MKL-2 cell lines were highly sensitive to TTI-3114, with a mean IC_50_ of around 10 nM and 51.6 nM, respectively. The MCC13 and MKL-1 cells were the less sensitive, with calculated IC_50_ values of 23 μM for MCC13 and 70.1 μM for MKL-1 (Figure 1B). These results display that MeSG induces cytotoxicity in human Merkel carcinoma cell lines. 

### 3.2. MeSG Efficiently Impairs Migration and Invasion of Virus-Positive MCC (VP-MCC) Cells

**Figure 2:**
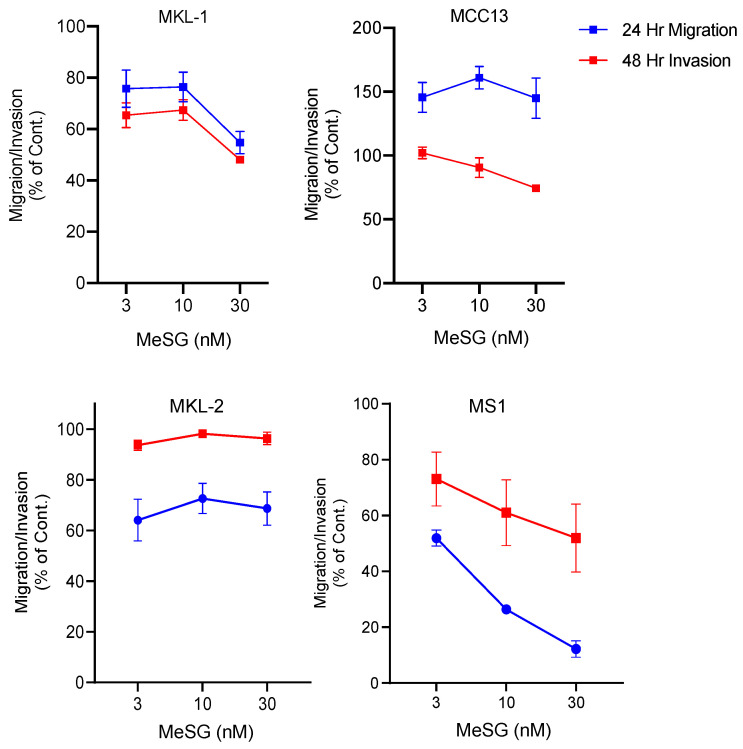
MeSG inhibits cell migration and invasion of MCC cell lines. VP-MCC (MS-1, MKL-1, MKL-2) and VN-MCC (MCC13) cells were seeded on a polycarbonate membrane (migration) or a polycarbonate membrane coated with a dried basement membrane matrix solution (invasion). Cells were allowed to migrate (24 h) or invade (48 h) toward 10% or 20% FBS in the presence or absence of 0, 3, 10, or 30 nM MeSG. Migrated or invaded cells on the bottom of the membrane were stained and quantified. At least three independent samples were analyzed (mean ± SEM).

MCC is known to metastasize early and aggressively to both local and systemic locations [43]. This metastatic nature of the disease may be promoted by expression of the MCPyV sT antigen [44,45]. Schweinfurthins induce morphological changes and actin cytoskeleton rearrangements [46,47,48]. However, the impact of schweinfurthins on cell migration and invasion has yet to be explored. The effect of MeSG on migration and invasion in MKL-1, MKL-2 and MS-1 (MCPyV^+^), and MCC13 (MCPyV^−^) cell lines was investigated through the use of polycarbonate membrane transwell migration and invasion assays. Cells were allowed to migrate for 24 h or invade for 48 h in the presence or absence of MeSG. Migration and invasion of the virus-positive MKL-1 and MS-1 cells were reduced upon incubation with MeSG. For the MKL-2 cells, only cell migration was impaired upon MeSG treatment, whereas invasion was not affected. Additionally, MeSG treatment did not appear to affect cell migration or invasion of the virus-negative MCC13 cells (Figure 2).

### 3.3. MeSG Induces Cell Death in MCC

Schweinfurthins have previously been shown to induce apoptosis following treatment in human glioblastoma cancer cells [46]. To determine if the decrease in MCC viability following MeSG treatment was due to induction of apoptosis or necrosis, flow cytometry analysis with PE annexin V and 7-aminoactinomycin D (7-AAD) staining was performed. The effect of MeSG was compared to a low concentration of cisplatin, a well-known FDA-approved chemotherapeutic shown to induce apoptosis through several mechanisms [49]. PE annexin V and 7-AAD can differentiate between early apoptotic, late apoptotic and necrotic cells as early-stage apoptotic cells will stain positive for PE annexin V but exclude 7-AAD. Necrotic cells will have a population of only 7-AAD positive cells. 

**Figure 3:**
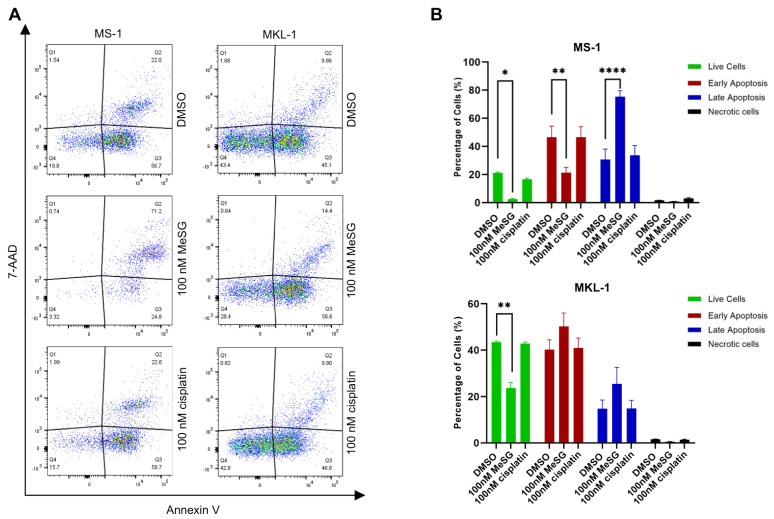
Treatment with MeSG induces cell death of MCC cell lines. (**A**) MeSG is more potent than cisplatin in inhibiting the growth of MCC in vitro. MS-1 and MKL-1 cells were incubated with 100 nM MeSG or 100 nM cisplatin for 48 h. Cell apoptosis or necrosis was determined by flow cytometry with Annexin V and 7-AAD staining. MeSG treatment showed higher efficacy in MCCs compared to the same dose (100 nM) of cisplatin treatment. Data are representative of three independent experiments. (**B**) Treatment with MeSG leads to increased late apoptosis in MS-1 cells. Error bars represent SEM; *n* = 3. Data was analyzed using GraphPad Prism software (GraphPad Software, Inc., La Jolla, CA, USA). The one-way analysis of variance (ANOVA) was used to determine statistical significance (MS−1 graph, * *p*  = 0.0229, ** *p* = 0.0020, **** *p* < 0.0001 and MKL−1 graph, ** *p* = 0.00119).

MCPyV^+^ MKL-1 and MS-1 were incubated in the absence or presence of MeSG at a cytotoxic concentration (100 nM) for 48 h. Following treatment, apoptotic and necrotic markers were evaluated using multicolor flow cytometry. Interestingly, MS-1 cells treatment with MeSG induced late apoptotic cell death (annexin V^+^/7-AAD^+^) as observed by an increase in the percentage of cells in those stages (Figure 3). In MKL-1 cells, MeSG induced mild changes in cell death, detected in both early and late apoptotic stages (Figure 3). These results demonstrate that treatment with MeSG more effectively induces apoptosis in MCPyV^+^ MCC cell lines but also varies depending on tumor heterogeneity. 

### 3.4. MeSG Inhibits sT Antigen-Induced Cell Growth Activity

**Figure 4:**
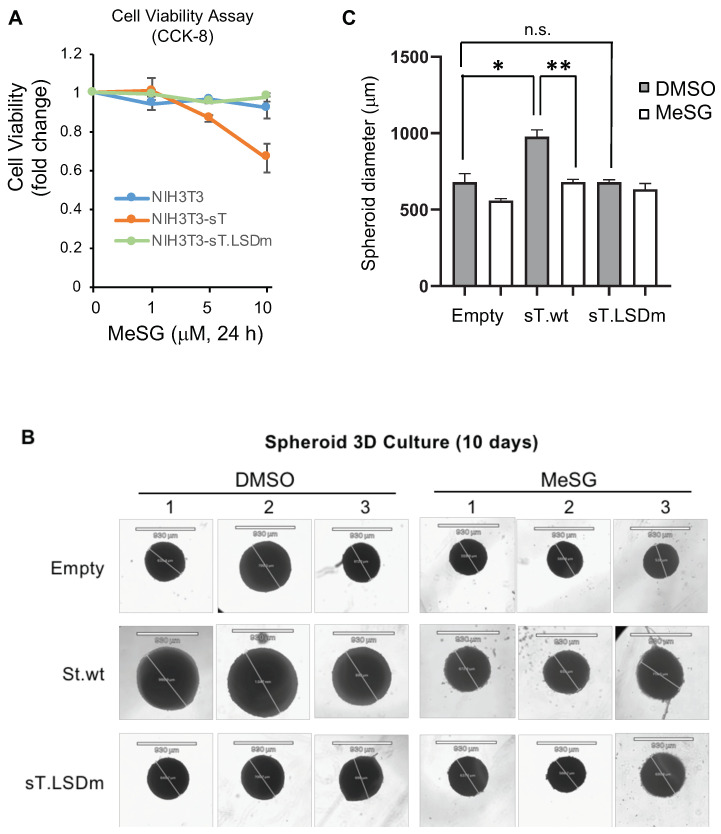
MeSG inhibits sT-induced cell proliferation. (**A**) Effects of MeSG on cell proliferation of NIH3T3 cells-expressing MCPyV sT were determined by CCK8 cell proliferation assay. NIH3T3 cells were transduced with lentivirus expressing empty vector, sT, or sT LSD mutant (st.LSDm) and treated with MeSG for 24 h. Small T-expressing cells are specifically sensitive to MeSG treatment, inhibiting cell growth. (**B**) Spheroids are generated in a cell-repellent tissue culture dish and treated with MeSG treatment (1 μM) for 10 days. The scale bar represents 930 μm. The spherical-shaped spheroids generally maintained their morphology over time. Images were taken with REVOLVE4 microscope and analyzed (**C**) using Echo Pro software (Echo Laboratories). Data was analyzed via one-way ANOVA (mean ± SEM, *n* = 3, * *p* < 0.05, ** *p* < 0.01, n.s.: not significant).

The small T antigen is the main oncogene in VP-MCC and drives cellular transformation in rodent fibroblast cells [26]. Since VP-MCC cells are more sensitive to MeSG treatment (Figure 1B), mouse NIH3T3 cells were transduced with an empty vector, sT, or sT LSD mutant (sT.LSDm) lentiviruses to determine if sT-induced transformation sensitizes cells to MeSG treatment as the LSD domain is required for sT-transforming activity [26]. MeSG treatment (5, 10 μM for 24 h) displayed selective toxicity against only wild-type sT-expressing cells (Figure 4A). The cell viability of empty vector or sT.LSDm-expressing cells were not affected by MeSG treatment, indicating that this drug is selectively targeting sT-expressing cells. 

Three-dimensional (3D) cell culture models, such as spheroids, can be used to develop new anticancer agents because they can closely mimic the main features of solid human tumors [50]. To analyze the therapeutic effect and tumor-penetrating capacity of MeSG as an anticancer therapeutic on VP-MCC, the 3D tumor spheroid proliferation analysis of MCPyV sT-expressing NIH3T3 cell line was performed. MeSG (1 μM) was added into the cell culture well after spheroid formation. The spheroids showed a significant delay of growth (~30% reduction in size) only in wild-type sT-expressing cell groups compared to control groups (empty vector and sT.LSDm) when exposed to 1 μM MeSG (single dose) for 10 days (Figure 4B,C).

### 3.5. MeSG Induces Transcriptome Changes in MS1 

To further elucidate the potential mechanism of the MeSG on MCC cell death, we performed RNA-seq analysis on MS-1 cells treated with DMSO or MeSG (100 nM) for 48 h. The raw gene counts were normalized in a standard distribution, and the basal expression difference was analyzed using the integrated Differential Expression and Pathway (iDEP) workflow [39]. K-means clustering and GO molecular function analysis revealed that cells treated with MeSG were upregulated in pathways associated with “MAP kinase phosphatase activity” and “phosphatidylinositol 3-kinase catalytic subunit binding” (Supplementary Materials). Notably, proteins enriched in the MAP kinase (MAPK) phosphatase activity pathway were dual-specificity phosphatases (DUSPs), which negatively regulate the MAPK pathway (Supplementary Table S1) [51]. 

**Figure 5:**
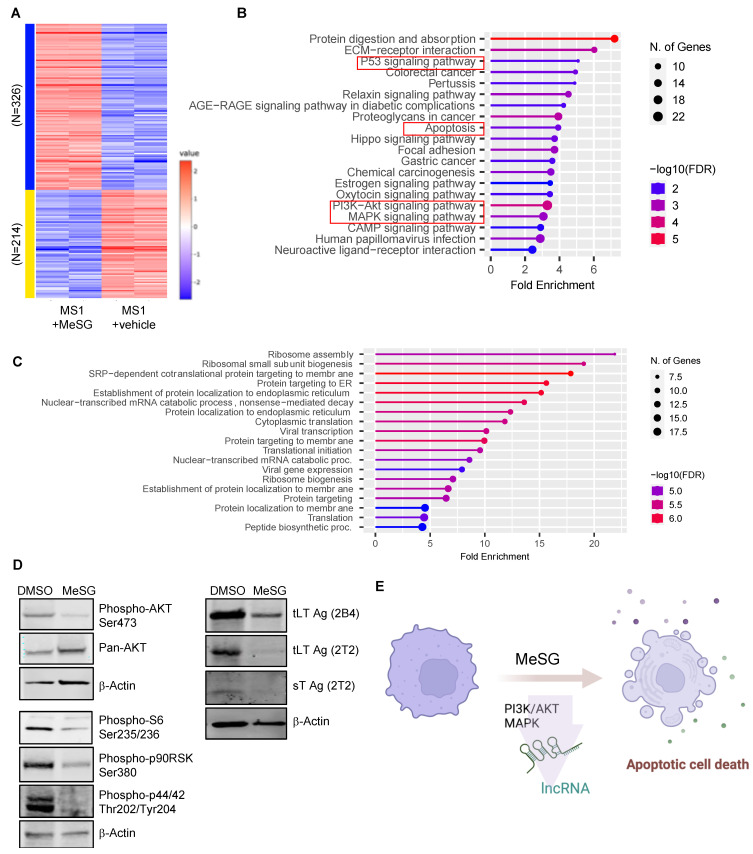
Different gene expression profile of MS-1 induced by MeSG. (**A**) RNA-seq and DEG analysis. Expression heatmap of DEGs between MeSG-treated (100 nM) and non-treated MS-1 control cells (+vehicle) is shown. (**B**) Results of the ShinyGO pathway enrichment analysis for the differentially expressed genes. P53, apoptosis, PI3K/AKT and MAPK signaling pathways were identified as potential biological processes associated with MeSG. (**C**) Gene changes in cancer cell lines (A549 and MS−1) treated by schweinfurthin analogs. (**D**) Analysis of PI3K/AKT and MAPK pathways in MS-1 treated with MeSG. MeSG (1 μM, 48 h) downregulates MCPyV T antigen proteins, phosphorylation of AKT, p44/42 (ERK1/2), p90RSK, and S6 ribosomal protein (the downstream target of both PI3K/AKT and MAPK) in MS-1. tLT: tumor-derived large T. (**E**) MeSG modulates the multiple pathways involved in PI3K/AKT and MAPK pathways to induce apoptotic cell death of MCC cell lines. The long noncoding RNAs (lncRNAs) may be essential in MCC progression.

DeSeq2 analysis revealed that MeSG treatment led to an upregulation of 326 differentially expressed genes (DEGs) and a downregulation of 214 DEGs (Figure 5A). These DEGs were run through ShinyGO for gene set enrichment analysis [40]. Notably, the results indicated that these DEGs were enriched in p53, apoptosis, PI3K-AKT, and MAPK signaling pathways (Figure 5B, Supplementary Table S1). Additionally, long noncoding RNAs (lncRNAs) that can function as either oncogenes or tumor suppressors [52] were found to be modulated by MeSG (Supplementary Table S1, Supplementary Materials). 

When compared to a previously reported dataset [41] in sensitive SF-295 and resistant A549 cells treated with the synthetic schweinfurthin analogue, TTI-3066, gene alterations were significantly correlated with the behavior of the more resistant cell line, A549 (Pearson: *p* = 0.001635, cor = 0.237; Spearman rank order: *p* = 0.01562, rho = 0.183; correlations insignificant for SF-295). ShinyGO analysis (GO Biological Process with FDR cutoff of 0.1) indicates that this correlation is driven by genes involved in ribosomal function and protein targeting (Figure 5C). Notably, four genes were significantly reduced in all three cell lines under treatment with schweinfurthins: two mitochondrial ribosomal proteins (MRPL11, MRPL12), NIP7 (involved in ribosomal biogenesis), and FARSB (Phenylalanyl-TRNA Synthetase Subunit Beta). These similarities suggest that schweinfurthin-treated cells may generally reduce translation in an effort to resist the effect of schweinfurthins or otherwise may be unable to translate new proteins as effectively as non-treated cells. Despite these genetic changes, it is clear that MS-1 cells cannot resist the effects of the schweinfurthins and ultimately undergo cell death processes. 

MeSG also significantly decreased T antigen protein levels, AKT kinase activity measured by AKT Ser473 phosphorylation, p90 RSK, p44/42 MAPK (ERK1/2), and S6 ribosomal protein phosphorylation in MS-1 (Figure 5D). Altogether, these data suggest that MeSG treatment on VP-MCC cells can regulate the PI3K/AKT and MAPK signaling pathways and lncRNAs to restrict cell growth and induce apoptosis, demonstrating the promise of schweinfurthin as a therapeutic agent for MCC (Figure 5E). 

## 4. Discussion

Natural and synthetic schweinfurthins are unique, potent, and selective cytotoxic compounds. However, their mechanism of action and differential sensitivity is still unknown [4]. Similar to what has been observed in other tumor types, the degree of sensitivity to schweinfurthins varies based on cell line [53]. Here, we show that the schweinfurthin analog MeSG impairs viability of human MCC cell lines to varying degrees. MS-1 cell line showed a high level of sT antigen expression compared to other cell lines [54], an interesting observation by which expression of the MCPyV sT antigen confers sensitivity to MeSG. MCPyV sT oncogene expression is known to activate MAPK [55] and PI3K/AKT/mTOR signaling for its transforming activity [26], consistently supporting our results and importance of these pathways in MCC progression. MeSG appears to induce cytotoxicity through an apoptotic pathway as detected by an increase in Annexin V, although the precise mechanism of action on viral antigens needs further investigation using xenograft models of virus-positive and -negative MCCs. 

Dysregulation of PI3K/AKT/mTOR signaling frequently occurs in many human diseases, including MCCs. Several preclinical data support the efficacy of PI3K/AKT/mTOR pathway inhibition in MCC-derived cell lines [56,57,58]. Though MeSG is known to negatively regulate PI3K/AKT/mTOR signaling pathway [36], the efficacy of MeSG could be limited due to drug resistance, especially in PTEN-deficient cancer cells [59]. Activation of AKT by the loss of PTEN, an inhibitor of the PI3K/AKT signaling pathway, was frequently observed in MCC [22,60,61] and could likely limit the clinical applications. Especially since previous studies have shown that MKL-1, MKL-2, and MCC13 are more resistant to PI3K inhibitors compared to other MCC cell lines such as Waga and Peta [56,62]. Overall, mTOR pathway inhibitors alone are primarily cytostatic, thus they are more synergistic in combination with other targeted chemotherapeutic approaches to induce apoptosis of these cancer cells [63]. Although more detailed information on the dose-response effects of combinations will be required in drug combination therapy applications, ’pharmaceutical’ multimodal treatments could be the suggested path for cancers with higher resistance like MCCs to increase treatment efficacy of MeSG. 

MeSG also inactivated ERK/MAPK pathway molecules as shown by the loss of p90RSK, ERK1/2, and S6 ribosomal protein phosphorylation in MS-1 (Figure 5D). These findings corroborate our RNA-seq data, indicating that MeSG treatment can negatively regulate both PI3K/AKT and MAPK/ERK pathways, potentially through the upregulation of DUSPs (Supplementary Table S1) [51], and dysregulate ribosomal biogenesis.

Despite considerable development in our understanding of lncRNA over the past decade, only a fraction of annotated lncRNAs has been examined for biological function. Recent findings reveal diverse functions for lncRNA involved in the mTOR signaling regulation [64]; thus, lncRNAs have emerged as therapeutic targets due to their ability to modulate multiple pathways in regulation of proteins or RNA molecules in human cancers [65]. Our RNA-seq data analysis also shows intriguing aspects of a changing lncRNA pool in MS-1 cells treated with MeSG. Specifically, oncogenic lncRNAs frequently associated with cancer metastasis, such as H19 [66], LINC01134 [67] were significantly reduced in MS-1 cells by MeSG treatment, indicating the potential importance of lncRNA expression in MCC tumor progression. Certainly, developing clinical trials using the distinctive features of lncRNAs profiling will more likely emerge in cancer studies. 

In summary, here we investigated the therapeutic potential of schweinfurthin analog, MeSG in MCC cell lines in vitro. This compound selectively inhibited proliferation of the MCPyV sT-expressing mouse fibroblast cell line, implicating the promising antitumor activity of MeSG in VP-MCC and potentially other human skin cancers such as melanoma with activated MAPK and PI3K/AKT signaling [10], which will open new opportunities for clinical research. A number of potential MCC therapeutics targeting critical cell signaling pathways including MAPK, PI3K/mTOR, bromodomain and extra-terminal domain (BET) family proteins, and B-cell lymphoma 2 (BCL-2) inhibitors (navitoclax) were reported to either induce cell death in MCC cells or potentially target MCPyV-infected cells [56,68,69,70,71,72]. The discovery of a previously undefined genetic signature in MCCs, combined with novel therapeutics research, will advance our understanding of the molecular mechanisms of MCC pathogenesis and enable the development of novel interventional strategies for this aggressive cutaneous malignancy, for which few treatment strategies are currently available. 

## Data Availability

All data are available from the corresponding authors upon reasonable request.

## Supplementary Materials

The following supporting information can be downloaded at: https://www.mdpi.com/article/10.3390/v14091848/s1. Table S1: Enriched pathways in MS-1 associated with MeSG treatment.
